# A novel tumor purity and immune infiltration-related model for predicting distant metastasis-free survival in prostate cancer

**DOI:** 10.1186/s40001-023-01522-8

**Published:** 2023-11-28

**Authors:** Qiang Su, Yongbei Zhu, Bingxi He, Bin Dai, Wei Mu, Jie Tian

**Affiliations:** 1 School of Engineering Medicine, Beihang University, Beijing, 100191 China; 2 Key Laboratory of Big Data-Based Precision Medicine (Beihang University), Ministry of Industry and Information Technology, People’s Republic of China, Beijing, 100191 China; 3 Clinical Laboratory Medicine, Beijing Shijitan Hospital, Capital Medical University, Beijing, 100038 China; 4 Neurosurgery department, Beijing Shijitan Hospital, Capital Medical University, Beijing, 100038 China; 5 CAS Key Laboratory of Molecular Imaging, Beijing Key Laboratory of Molecular Imaging, Institute of Automation, Chinese Academy of Sciences, Beijing, 100190 China

**Keywords:** Prostate cancer, Tumor purity, Immune infiltration, Distant metastasis-free survival, Nomogram

## Abstract

**Background:**

umor cells, immune cells and stromal cells jointly modify tumor development and progression. We aim to explore the potential effects of tumor purity on the immune microenvironment, genetic landscape and prognosis in prostate cancer (PCa).

**Methods:**

Tumor purity of prostate cancer patients was extracted from The cancer genome atlas (TCGA). Immune cellular proportions were calculated by the CIBERSORT. To identify critical modules related to tumor purity, we used weighted gene co-expression network analysis (WGCNA). Using STRING and Cytoscape, protein–protein interaction (PPI) networks were constructed and analyzed. A Gene Ontology (GO), Kyoto Encyclopedia of Genes and Genomes (KEGG) pathway, Disease Ontology (DO), and Gene Set Enrichment Analysis (GSEA) enrichment analysis of identified modules was conducted. To identify the expression of key genes at protein levels, we used the Human Protein Atlas (HPA) platform.

**Results:**

A model of tumor purity score (TPS) was constructed in the gene expression omnibus series (GSE) 116,918 cohort. TCGA cohort served as a validation set and was employed to validate the TPS. TPS model, as an independent prognostic factor of distant metastasis‐free survival (DMFS) in PCa. Patients had higher tumor purity and better prognosis in the low-TPS group. Tumor purity was related to the infiltration of mast cells and macrophage cells positively, whereas related to the infiltration of dendritic cells, T cells and B cells negatively in PCa. The nomogram based on TPS, Age, Gleason score and T stage had a good predictive value and could evaluate the prognosis of PCa metastasis. GO and KEGG enrichment analyses showed that hub genes mainly participate in T cell activation and T-helper lymphocytes (TH) differentiation. Hub genes were mainly enriched in primary immunodeficiency disease, according to DO analysis. SLAMF8 was identified as the most critical gene by Cytoscape and HPA analysis.

**Conclusions:**

Dynamic changes in the immune microenvironment associated with tumor purity could correlate with a poor DMFS of low-purity PCa. The TPS can predict the DMFS of PCa. In addition, prostate cancer metastases may be related to immunosuppression caused by a disorder of the immune microenvironment.

**Supplementary Information:**

The online version contains supplementary material available at 10.1186/s40001-023-01522-8.

## Introduction

Prostate cancer (PCa) is the primary malignancy among men, responsible for 14.1% of new cases, and ranks 5th in terms of cancer-related deaths (with a mortality rate of 6.8%) worldwide [1]. In China, prostate cancer accounts for 8.16% of new cancers in men (ranking as the 6th most common malignancy), with a mortality rate of 13.61 (ranks 7th in terms of cancer-related deaths) [2]. The leading cause of death is metastasis in PCa. The outcome of metastatic PCa is inferior, as only 30% of patients could survive for 5 years [3]. Gleason score and tumor, node, metastasis (TNM) stage are prognostic factors. Unfortunately, there may be vast differences in clinical outcomes between patients with the same Gleason score, making it essential to identify critical factors influencing prognosis.

It has been found that tumor purity is significantly related to the clinical characteristics and genetic features of patients with tumors. It is possible to develop systematic biases in recurrence risk, tumor genotyping and efficacy prediction by ignoring the influence of tumor purity [4]. A low-purity tumor sample has a higher mutational burden and more immune cells. Immune cells’ inflammatory response may result in tumor cells mutating more rapidly, which may improve the effectiveness of immunotherapy [4]. Previous studies have indicated that tumor purity is one way of determining the efficacy of immunotherapy. Gastric and colon cancer prognosis has been demonstrated to positively correlate with tumor purity [5, 6]. However, few studies have considered the influence of tumor purity in the prognosis of PCa.

A tumor purity calculation was performed using the ESTIMATE algorithm in this study [7]. The CIBERSORT algorithm was used to verify further whether low- and high-purity tumors had significantly different immune cell infiltration levels. After that, the tumor purity co-expression network was constructed using weighted gene co-expression network analysis (WGCNA) [8]. The co-expression modules contained genes that were most related to tumor purity. Gene signatures associated with distant metastasis-free survival (DMFS) of prostate cancer were identified using the least absolute shrinkage and selection operator (LASSO)–COX regression analysis. Then, tumor purity score (TPS) was constructed. Kaplan–Meier and receiver operating characteristics curve (ROC) analyses indicated that PCa patients with higher TPS had worse prognoses. A nomogram was created using TPS and clinical parameters. In addition, all hub genes underwent Gene Ontology (GO), Kyoto Encyclopedia of Genes and Genomes (KEGG), and Disease Ontology (DO) enrichment analysis. Our study has revealed a relationship between tumor purity and immune cell infiltration in PCa and built a robust predictive model for clinical application.

## Materials and methods

### Data acquisition

The gene expression omnibus (GEO) database was created by the National Center for Biotechnology Information (NCBI), which contains gene expression data submitted by research institutes around the world [9]. The cancer genome atlas (TCGA) was launched by the National Cancer Institute (NCI) and the National Human Genome Research Institute (NHGRI) in 2006. More than 20.000 samples data from 33 types of cancer were contained in the TCGA database, including transcriptome data, genomic variation data, methylation data, and clinical data. The selection criteria for public data sets were as follows: (1) available transcriptome (microarray or RNA sequencing data; (2) available information on basic clinicopathological parameters and metastatic survival; (3) a sample size of greater than 200. Therefore, gene expression omnibus series (GSE) 116,918 array expression data and TCGA sequencing data of PCa were screened [10, 11]. Clinicopathological information was collected from different portals: TCGA (https://portal.gdc.cancer.gov/) and GEO (https://www.ncbi.nlm.nih.gov/geo/). Clinicopathological characteristics of the enrolled patients in detail for both data sets were described in Table 1.

**Table 1:** Clinical and pathological characteristics of TCGA and GEO data sets

Characteristics	TCGA (*n* = 495)	GSE116918 (*n* = 248)
Age, *n* (%)		
≤ 60	222(44.8)	35(14.1)
> 60	273(55.2)	213(85.9)
PSA, *n* (%)		
≤ 10	–	50(20.2)
> 10	–	198(79.8)
T stage, *n* (%)		
T1	–	51(20.6)
T2	187(37.8)	76(30.6)
T3	291(58.8)	92(37.1)
T4	10(2.0)	4(1.6)
NA	7(1.4)	25(10.1)
N stage, *n* (%)		
N0	344(69.5)	–
N1	78(15.8)	–
NA	73(14.7)	–
Gleason score, *n* (%)		
< 7	45(9.1)	42(17.0)
= 7	246(49.7)	99(39.9)
> 7	204(41.2)	107(43.1)
MET, *n* (%)		
No	444(89.7)	226(91.1)
Yes	17(3.4)	22(8.9)
NA	34(6.9)	–

*PSA,* prostate-specific antigen; *MET*, metastasis; *NA,* not applicable

### Immune infiltration and tumor purity calculation

The ESTIMATE package in R software was performed to calculate stromal, ESTIMATE and immune scores in malignancy tissues [7]. To determine the tumor purity of each malignancy tissue in TCGA-Prostate Adenocarcinoma (PRAD), the ESTIMATE algorithm was used. According to the median tumor purity, we categorized prostate cancer patients into low- and high-tumor purity groups. The infiltration level of immune cells was evaluated by Single-sample GSEA (ssGSEA) analysis, which was achieved using the Gene Set Variation Analysis (GSVA) package [12]. The difference in infiltration levels of LM22 human immune cell subtypes was evaluated with the CIBERSORT algorithm between low- and high-tumor purity groups [13].

### Differential gene screening

In low- and high-tumor purity groups, TCGA-PRAD transcriptome files were subjected to differentially expressed genes (DEGs) analysis using the R package “Limma” [14]. Genes with a *p* value < 0.05 (false discovery rate (FDR) correction, empirical Bayesian modulation method in Limma R package) and log2 fold change ≥ 2 were selected as DEGs.

### WGCNA analysis

WGCNA was performed to identify a set of tumor purity-related co-expressed genes in prostate cancer [8]. In this study, we set an R square of 0.9, a soft threshold of 7, and a minimum gene module of 50, generating 15 non-gray modules. The similarities and differences between all modules were calculated. The module can be used to construct a dendrogram, enabling the identification of key differential genes that exhibit the strongest correlation with tumor purity.

### Prognostic model based on LASSO-COX

To screen characteristic variables related to the survival of patients with metastatic prostate cancer in the key differential genes, the LASSO-COX regression classification model was constructed in GSE116918 set using the “glmnet algorithm” package in R software [15, 16]. Then, TPS was calculated using the sum of LASSO coefficients multiplied by the expression value of each gene in both sets.

### ROC and survival analysis

According to the median TPS, patients were divided into low- and high-TPS groups in the GSE116918 data set. The "survival" R package was performed to analyze the DMFS of patients in two groups (https://CRAN.R-project.org/package=survival). To quantify an area under the curve (AUC), the R package "survival ROC" was performed to depict a time-dependent ROC plot [17]. The predictive ability of TPS was verified by predictive accuracy and survival difference in the TCGA data set.

### Univariate and multivariate COX models

To screen independent risk elements for PCa, univariate and multivariate Cox regression analyses were implemented using the "survival" R package (https://CRAN.R-project.org/package=survival). Exclusion criteria were incomplete data, such as survival status and clinical variables. In the GSE116918 data set, 223 patients had complete information. Clinical variables included age, PSA, T stage, and Gleason. In the TCGA data set, 391 patients had complete information. Clinical variables included PSA, T stage, N stage, and Gleason.

### Nomogram analysis

Based on clinical variables and TPS, a prediction model was developed and constructed as a nomogram. The nomogram was constructed using "foreign", "survival" and "rms" R packages (https://CRAN.Rproject.org/package=foreign, https://CRAN.R-project.org/package=survival, https://CRAN.R-project.org/package=rms). The calibration chart was implemented to assess the performance characteristics of this nomogram. The training (GSE116918) data set was used to build a nomogram model for DMFS prediction. As a validation data set, the TCGA cohort was then used to validate the model.

### Identification and validation of hub genes

According to the tumor purity, differential genes with a *p* value under 0.05 and a genetic significance greater than 0.8 in green module were selected as the hub genes. A heat map and box map of hub genes expression were drawn according to the tumor purity group through the package ggpubr (https://CRAN.R-project.org/package=ggpubr), and pheatmap (https://CRAN.R-project.org/package=pheatmap). Make a correlation graph for the hub genes and tumor purity using the corrplot package (http://CRAN.R-project.org/package=corrplot).

### Developing and analyzing protein–protein interaction (PPI) networks of hub genes

To estimate protein interactions between hub genes, the PPI network was constructed using the online database of the Search Tool for the Retrieval of Interacting Genes (STRING; http://string-db.org/) and significant differences were determined by a combined-score greater than 0.4 [18]. In addition, Cytoscape (version 3.8.2) was used to visualize the network [19].

### Analyses of gene ontology (GO), kyoto encyclopedia of genes and genomes (KEGG), and disease ontology (DO) enrichment

Separate analyses were conducted on hub genes based on GO, KEGG, and DO enrichment. To perform an enrichment analysis, filter conditions were set as follows: *p* value < 0.05, *q* value < 0.05, and enrichment results with significance if both conditions were met. To visualize enrichment analysis results and map bubble charts, we use the 'clusterProfiler' [20], the 'DOSE' [21], the 'enrichplot' and 'ggplot2' packages [22]. We visualized the first ten enrichment pathways created by GSEA enrichment analysis on hub genes.

### Verification of hub and TPS genes

First, we compared the expression of the top 20 hub genes and TPS genes in tumor tissues and normal tissues in the TCGA database. Second, the differentially expressed genes were paired and compared between tumors and adjacent normal tissues. Lastly, the Human Protein Atlas (HPA) database (https://www.proteinatlas.org/, accessed on 08 October 2023) was used to retrieve prostate tissue images of differentially expressed genes.

### Statistical analysis

Statistical analysis was implemented using R 3.6.3 software (https://www.r-project.org/). Using Spearman correlation analysis, the association between continuous variables was evaluated. The nearest neighbor estimation (NNE) method was performed to draw DMFS plots. To compare the difference in DMFS between the low-TPS and high-TPS groups, the log-rank test was used. For determining independent prognostic factors, univariate and multivariate Cox regressions were carried out, along with 95% confidence intervals (CI) and hazard ratios (HR). For comparison between the two groups, Wilcox tests were conducted. The *p* value < 0.05 was defined as statistically significant for all the analyses.

## Results

### Immune microenvironment and tumor purity

**Fig. 1:**
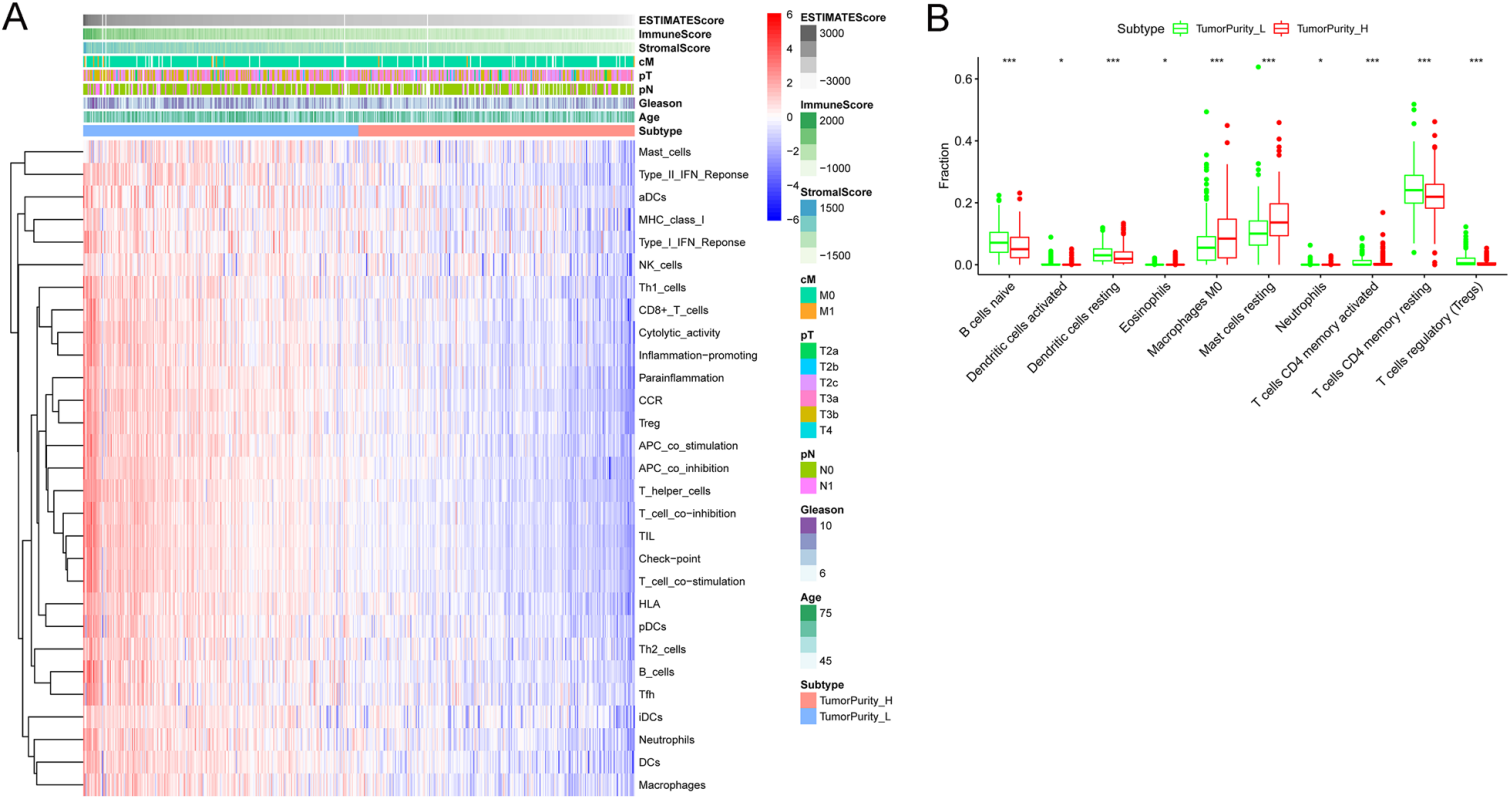
Tumor purity and immune cell infiltration in PCa. **A** Compared to the low-tumor purity group, there is a significantly different in immune cell infiltration and clinical features in the high-tumor purity group. **B** Comparing immune cells’ proportion between two tumor purity subgroups by CIBERSORT algorithm. *p value < 0.05; **p value < 0.01; ***p value < 0.001

The tumor purity of each PCa sample was evaluated using the ESTIMATE algorithm. As shown in Additional file 1: Table S1, immunological scores and tumor purity were calculated for each patient in the TCGA-PRAD data set. The distribution of clinical features and immune cell infiltration in low- and high-tumor purity groups were visualized using heat maps (Fig. 1A). There was a significant increase in immune cell infiltration in the low-purity group. In low- and high-tumor purity groups, the CIBERSORT algorithm was performed to calculate the differences in the contents of immune cells (Fig. 1B). The contents of B cells, CD4^+^ T lymphocytes, neutrophils, dendritic and eosinophils cells were significantly increased in the low-purity group. This verified the robustness of the ESTIMATE algorithm.

### Tumor purity and clinical features

**Fig. 2:**
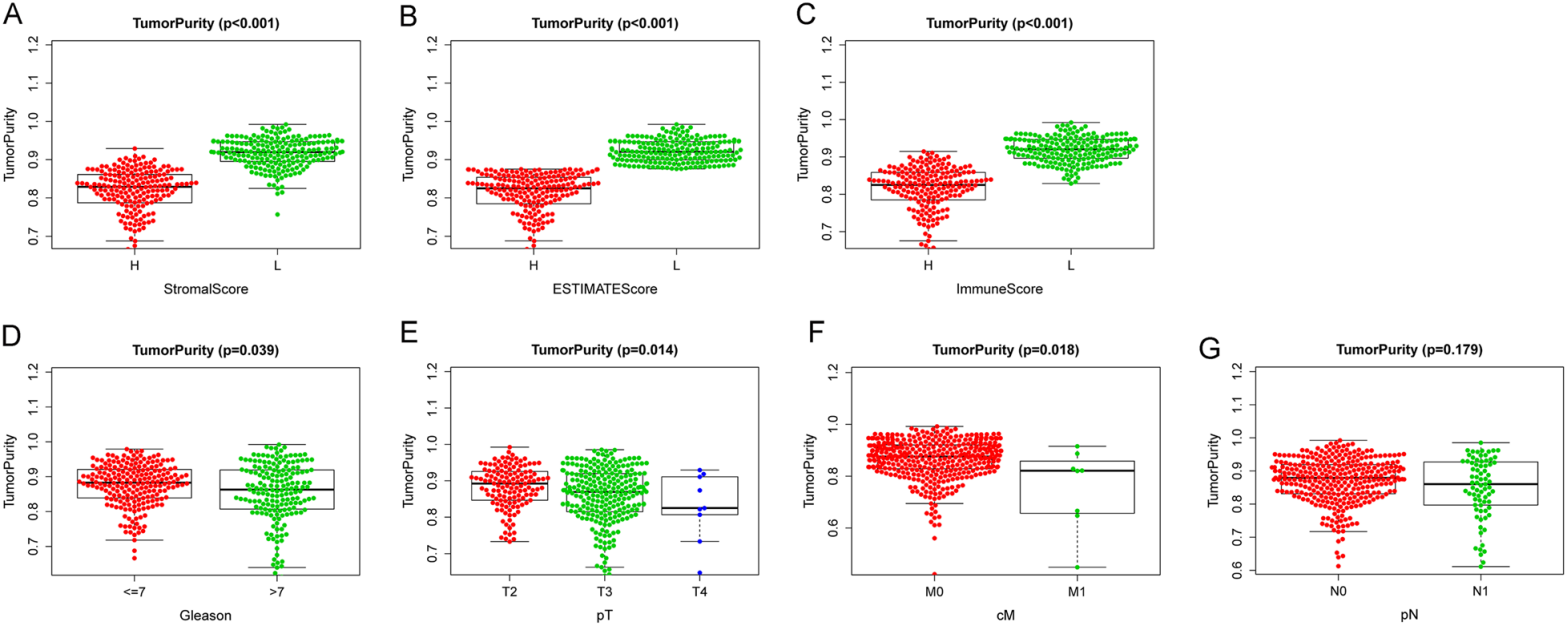
Tumor purity’s correlation with immune signatures and clinical features. **A** Stromal score. **B** Estimate score. **C** Immune Score. **D** Gleason. **E** Pathological T stage. **F** Clinical M stage. **G** Pathological N stage

Our study was conducted to examine the potential effects of tumor purity on clinicopathological features in PCa, as shown in Fig. 2. Tumor purity was significantly related to immune score, stromal score, ESTIMATE score, and clinical characteristics (*P* < 0.001). With an increase in Gleason (*p* = 0.039), T stage (*p* = 0.014) or M stage (*p* = 0.018), tumor purity was decreased significantly. However, there was no considerable decrease in tumor purity among patients with lymphatic metastasis (*P* = 0.179).

### Development of tumor purity score (TPS)

**Fig. 3:**
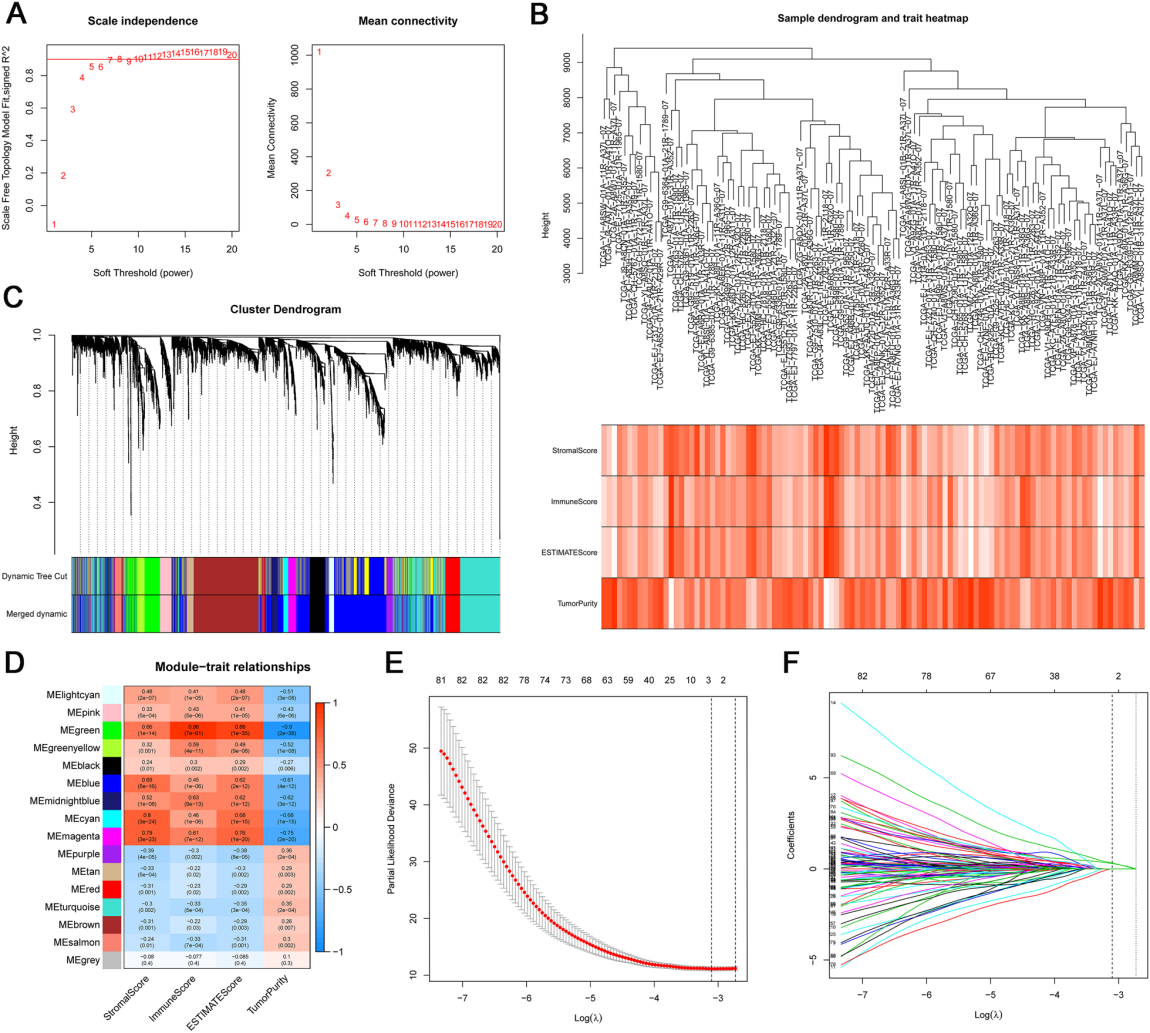
Tumor purity score model construction by WGCNA and Lasso COX analysis. **A** Analysis of soft thresholds. **B** Sample dendrogram and trait heatmap. **C** Merged dynamic gene cluster dendrogram. **D** Identification of tumor purity score-related gene clusters. **E, F** Construction of tumor purity score by Lasso Cox analysis

Scale-free R2 was defined as 0.9, with soft threshold power set to 7 (Fig. 3A). Sample dendrogram and trait heatmap was built (Fig. 3B). Using WGCNA analysis of 5379 differential genes, 15 co-expression networks were obtained (Fig. 3C), where each color represents a co-expression network. In addition, we identified 363 genes in the green module that have the strongest association with tumor purity (*r* = − 0.9, FDR = 10−38) using co-expression networks (Fig. 3D). We have uploaded Additional file 2: Table S2 with the WGCNA results. There were 282 genes shared between the TCGA and GSE116918 data sets for subsequent analysis. In the training set GSE116918, two genes (FCER1G and OLR1) were screened as signature genes by Lasso-COX (Fig. 3E, F). TPS was calculated according to the formula (TPS = FCER1G × 0.32572 + OLR1 × 0.29642).

### High TPS conferred a worse prognosis in PCa

**Fig. 4:**
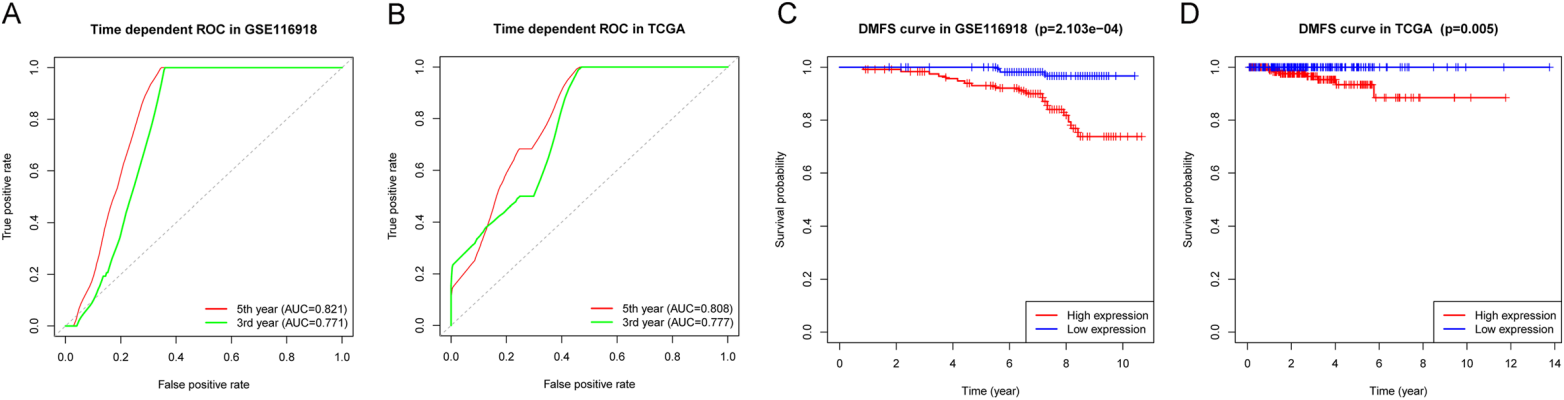
Tumor purity score model with high accuracy in predicting DMFS in PCa. **A**, **B** Time-dependent ROC plots were developed for the TPS model to predict the probability of DMFS after three and five years, in the training and validation data sets, **C**, **D** Based on the training and validation data sets, Kaplan–Meier survival analysis was performed on the DMFS according to TPS level

To verify TPS’s prediction ability, a ROC curve was drawn to calculate an AUC value in both training and validation sets. TPS had an excellent prediction effect. The AUC values in the training set were 0.821 for 5 years DMFS and 0.771 for 3 years DMFS (Fig. 4A). The AUC values in the validation set were 0.808 for 5 years DMFS and 0.777 for 3 years DMFS (Fig. 4B). PCa patients with higher TPS had shorter DMFS (*p* = 2.103–04, Fig. 4C). Fortunately, this finding was confirmed in the validation set (*p* = 0.005, Fig. 4D). For both training and validation data sets, multivariate and univariate COX models indicated that TPS was an independent prognostic factor, as shown in Tables 2 and 3.

**Table 2:** Univariate and multivariate Cox regression analyses of clinicopathologic features and TPS in the GSE116918 data set

Variables	Univariate Cox analysis		Multivariate Cox analysis	
	HR (95% CI)	*P*	HR (95% CI)	*P*
Age (years)				
≤ 60	1.000 (reference)	NA	1.000 (reference)	NA
> 60	1.140(0.337 − 3.855)	0.832	1.713(0.478 − 6.139)	0.409
PSA				
≤ 10	1.000 (reference)	NA	1.000 (reference)	NA
> 10	1.159(0.392 − 3.427)	0.790	0.835(0.258 − 2.704)	0.763
T stage	1.734(0.957 − 3.142)	0.070	1.182(0.614 − 2.276)	0.617
Gleason				
≤ 7	1.000 (reference)	NA	1.000 (reference)	
> 7	5.041(0.678 − 37.503)	0.114	2.186(0.269 − 17.761)	0.464
TPS	19.391(5.274 − 71.292)	< 0.05*	16.281(3.781 − 70.104)	< 0.05*

*PSA* prostate-specific antigen, *TPS* tumor purity score, *CI* Confidence intervals, *HR* hazard ratio, *NA* not applicable, * p value < 0.05, ** p value < 0.01, *** p value < 0.001

**Table 3:** Univariate and multivariate Cox regression analysis of clinicopathologic features and TPS in the TCGA data set

Variables	Univariate Cox analysis		Multivariate Cox analysis	
	HR (95% CI)	*P*	HR (95% CI)	*P*
Age (years)				
≤ 60	1.000 (reference)	NA	1.000 (reference)	NA
> 60	1.385(0.331–5.799)	0.656	1.088(0.230–5.139)	0.915
T stage	5.929(1.434–24.519)	0.014*	2.646(0.401–17.433)	0.312
N stage				
No	1.000 (reference)	NA	1.000 (reference)	NA
Yes	8.038(1.907–33.877)	0.005**	7.486(1.179–47.529)	0.033*
Gleason				
≤ 7	1.000 (reference)	NA	1.000 (reference)	NA
> 7	8.334(1.025–67.752)	0.047*	3.176(0.332–30.389)	0.316
TPS	1.065(1.028–1.103)	< 0.001***	1.082(1.033–1.134)	< 0.001***

*TPS* tumor purity score, *CI* confidence intervals, *HR* hazard ratio, *NA*: not applicable

*p value < 0.05; **p value < 0.01; ***p value < 0.001

### Nomogram development and validation

**Fig. 5:**
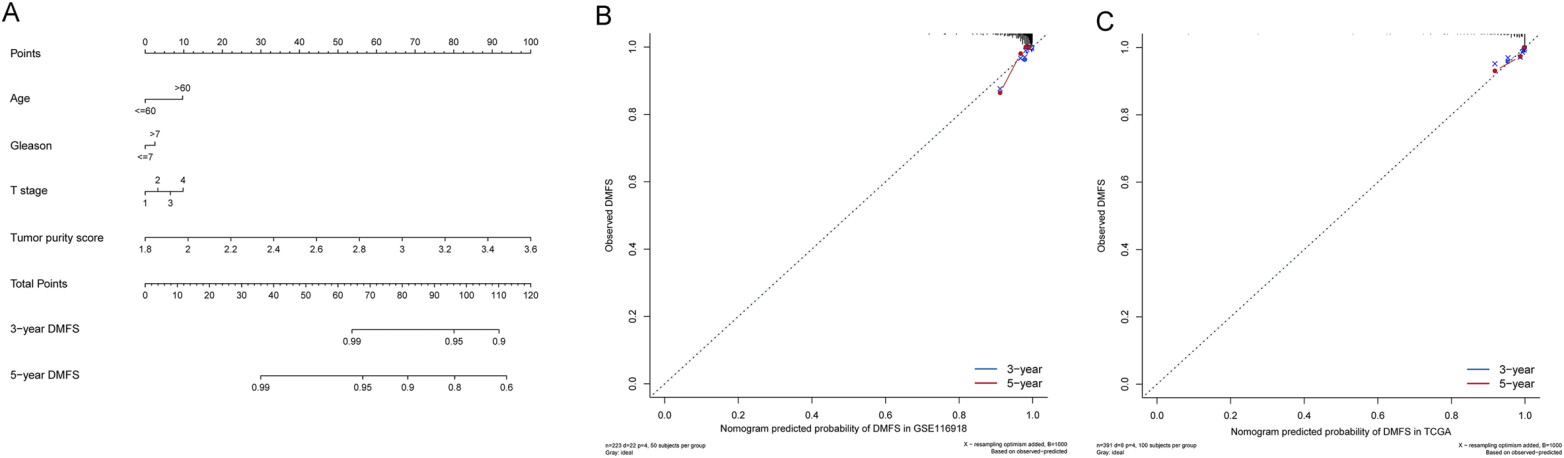
Development and validation of the nomogram. **A** Nomogram for predicting 3- and 5-year DMFS in PCa patients. **B** Calibration chart for the nomogram in the training set. **C** Calibration chart for the nomogram in the validation set

Nomogram was constructed using clinical features (age, Gleason and T stage) and TPS for the training set. Considering clinical features and TPS scores, the total score was calculated. The nomogram can be predicted from 3 to 5 years DMFS (Fig. 5A). In calibration plots, the predicted result was very close to the actual result. As for calibration plots, the nomogram was highly accurate in predicting the prognosis of PCa patients in both cohorts (Fig. 5B, C).

### Expression and correlation of hub genes

**Fig. 6:**
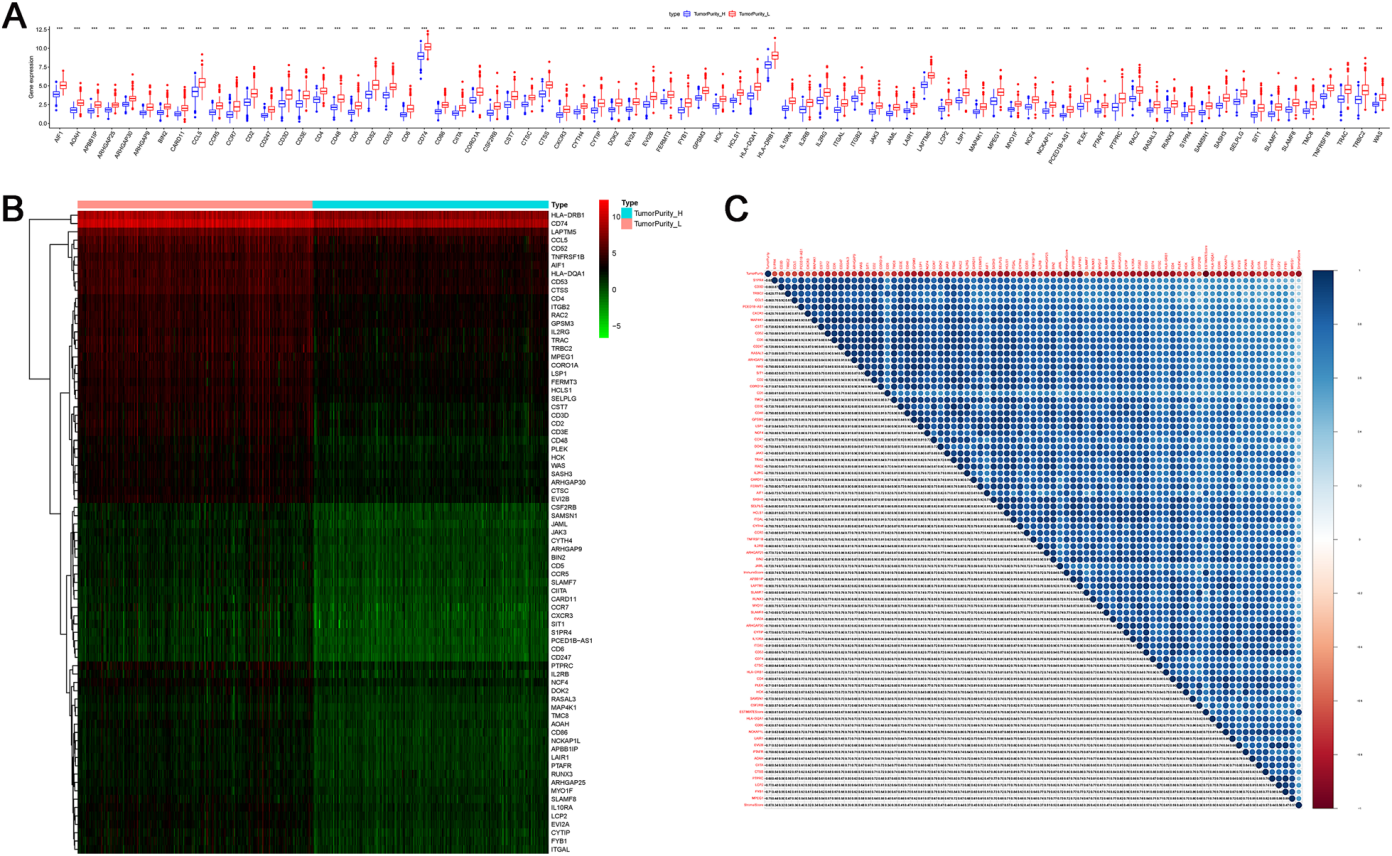
Hub genes differential expression and correlation analysis. **A** Hub genes with their *p* values in two tumor purity groups. **B** Heatmap of hub genes expression. **C** Correlation maps of immune score, stromal score, ESTIMATE score and hub genes. *p value < 0.05, **p value < 0.01, ***p value < 0.001

The expression of 77 hub genes was significantly different between the high and low tumor purity groups (Fig. 6A, B). In addition, significant correlations were found between the expression of 77 hub genes. There was a negative correlation between tumor purity and hub genes. The immune score, stromal score and ESTIMATE score were positively correlated with hub genes (Fig. 6C).

### PPI network module analysis

**Fig. 7:**
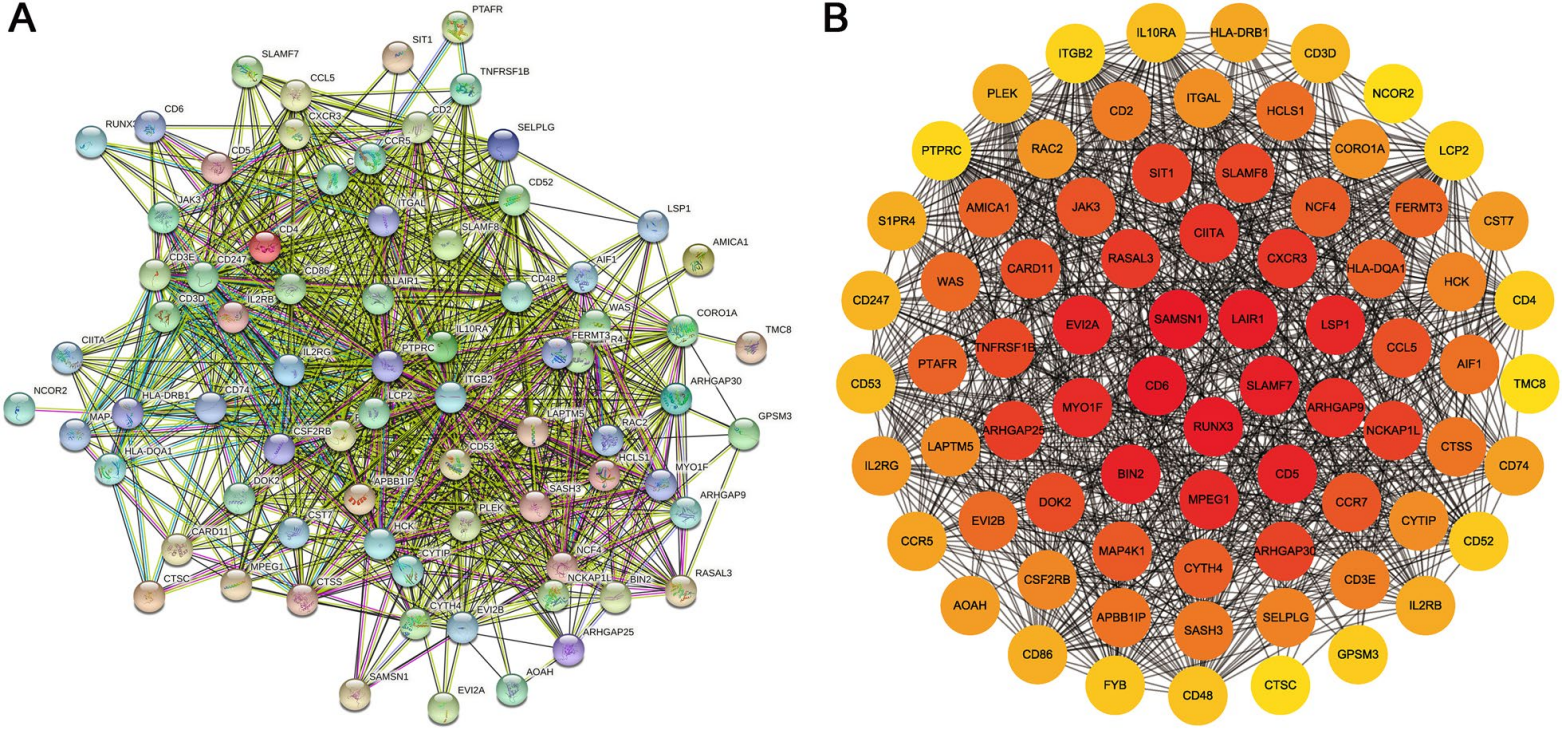
PPI Network Analysis of hub genes. **A** PPI network built using STRING database. In PPI network diagrams, nodes represent proteins and edges represent the interaction between proteins. **B** Visualization of PPI network by Cytoscape. Yellow to red represent increasing levels of Clustering Coefficient Ranking, i.e. yellow, low; orange, medium; red, high

To understand the biological meaning of the hub genes identified by the WGCNA analysis, a PPI network was constructed from 75 nodes and 1686 edges for these hub genes-encoding proteins, as shown in Fig. 7A. A PPI network graph was conducted by Cytoscape software using Clustering Coefficient Ranking method (Fig. 7B).

### Hub genes enrichment analysis

**Fig. 8:**
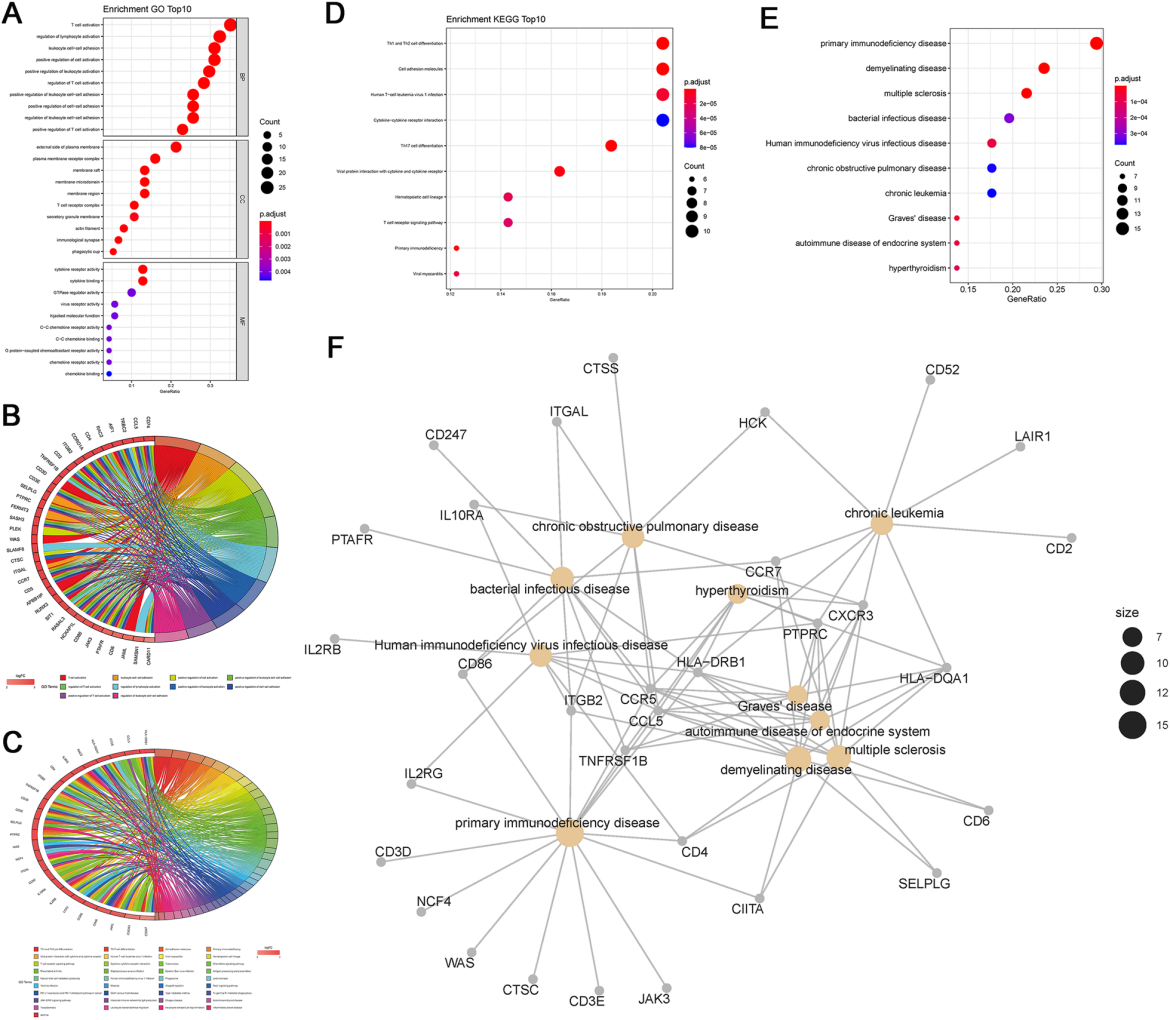
Enrichment analyses of hub genes were based on GO, DO, and KEGG in high and low- tumor purity group. **A** In the bubble plots of GO analysis, there were three major categories. **B** To visualize the top 10 biological process GO terms, a chord plot was employed. **C** In the bubble plots of KEGG analysis, there were top 10 KEGG enrichment pathway. **D** To visualize all the KEGG terms, a chord plot was employed. **E** In the bubble plots of DO analysis, there were top 10 DO enrichment pathway. **F** Graph illustrating the connections between the top 10 illnesses and hub genes

GO (gene ontology) enrichment results show T cell activation, regulation of lymphocyte activation, and leukocyte cell–cell adhesion are the major biological process (BP) involved by hub genes. Hub gene function results in a cellular component (CC) consisting mainly of the external side of plasma membrane, plasma membrane receptor complex, membrane raft, etc. Molecular functions (MF) of hub genes products include cytokine receptor activity, cytokine binding, and GTPase regulator activity, among others (Fig. 8A, B). According to the KEGG pathway enrichment analysis, hub genes were mainly involved in the Th1 and Th2 cell differentiation, followed by other pathways such as Cell adhesion molecules and Human T-cell leukemia virus 1 infection (Fig. 8C, D). In addition, the DO analysis reveals that hub genes were mainly enriched in primary immunodeficiency disease, ldemyelinating disease and omultiple sclerosis (Fig. 8E, F).

### Gene expression and immunohistochemistry stain in PCa

**Fig. 9:**
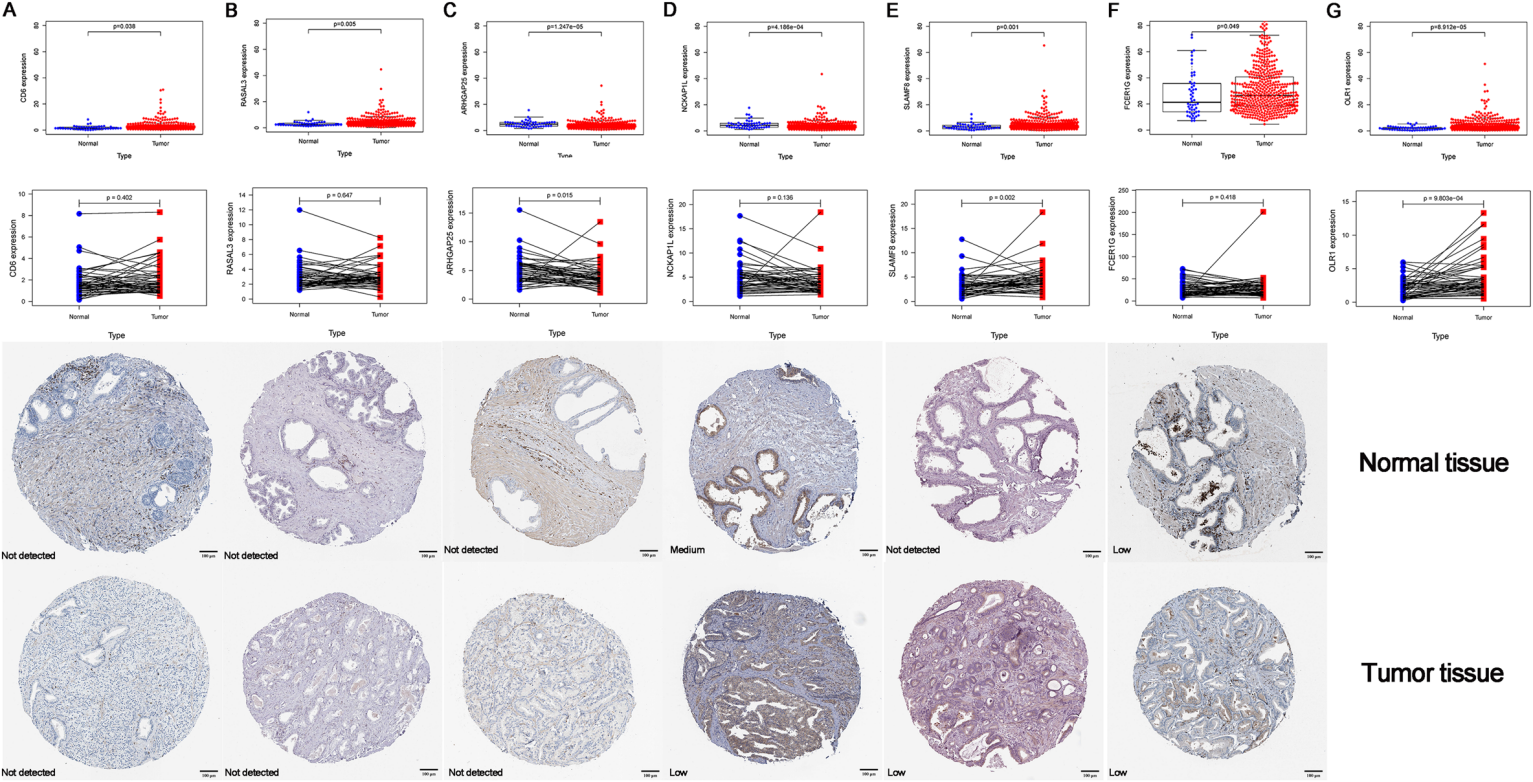
PCa clinical sample validation. **A** CD6, **B** RASAL3, **C** ARHGAP25, **D** NCKAP1L, **E** SLAMF8, **F** FCER1G, **G** OLR1 expression in TCGA and HPA databases. Normal and tumor tissues differed significantly in all these genes. Compared with paired adjacent normal tissues, SLAMF8 and OLR1 expression was significantly higher in tumor tissues, while ARHGAP25 expression was lower. Based on the HPA database, SLAMF8 protein expression was increased in tumor tissues compared to normal tissues

We validated the top 20 hub genes (SAMSN1, CD6, RUNX3, LAIR1, SLAMF7, BIN2, CD5, EVI2A, LSP1, MPEG1, MYO1F, ARHGAP9, CIITA, CXCR3, RASAL3, ARHGAP25, NCKAP1L, SIT1, ARHGAP30, SLAMF8) and TPS genes (FCER1G, OLR1) using clinical samples in TCGA database and representative immunohistochemistry (IHC) images from the Human Protein Atlas (HPA, https://www.proteinatlas.org/) database. Among these genes, CD6, RASAL3, ARHGAP25, NCKAP1L, SLAMF8, FCER1G and OLR1 were significantly different in PCa tissues. In addition, between tumor tissues and their paired adjacent normal tissues, ARHGAP25, SLAMF8 and OLR1 expression differed significantly. Even though ARHGAP25 showed decreased trends in the TCGA analysis, protein levels did not change significantly. According to the HPA database, prostate tissue was not immunohistochemically stained for OLR1. Finally, both protein and RNA levels of SLAMF8 showed significant increases in prostate tumor tissues (Fig. 9A–G).

## Discussion

Recently, with the development of precision therapy and immunotherapy for malignant tumors, an important role is played by the immune microenvironment in tumor metastasis, treatment response, and prognosis. In addition, tumor purity can reflect unique characteristics of the tumor microenvironment (TME) [4]. Meanwhile, the high morbidity and mortality of PCa make it a global public health problem [1, 2]. Therefore, our study focused on the tumor purity of PCa.

A tumor purity calculation was performed first in this study. By the median value of tumor purity, we divided PCa into low and high groups. We screened out differential genes and obtained key genes with the highest relationship with tumor purity by the WGCNA. The ESTIMATE R package, ssGSEA algorithm and CIBERSORT were performed to uncover TME landscapes of different tumor purity subgroups in PCa. To establish a TPS model relating to DMFS in prostate cancer, LASSO-COX regression was used. Using this model, DMFS of PCa can be predicted independently. An excellent accuracy nomogram that can predict three- and five-year DMFS for PCa patients has been developed and validated.

There was a substantial correlation between tumor purity and immune cell infiltration in prostate cancers, as well as clinical features. With an increase in Gleason score, T stage or M stage, tumor purity was decreased significantly. These results indicate that high tumor purity is related to a favorable outcome of PCa. The tumors with lower purity have a higher degree of malignancy and a worse prognosis. This is consistent with previous findings in gastric cancer [5], glioma [23] or colon cancer [6]. Furthermore, our conclusions are in general agreement with previous results showing that a low Gleason score is a good prognostic factor [24].

In recent years, computational tools have emerged in an endless stream, and tumor purity estimation methods based on different genetic data types have been proposed. The ESTIMATE algorithm and CIBERSORT algorithm adopted in this study can be used for RNA sequencing analysis [7, 13]. Our experiments are verified mutually in these two algorithms. The results generated by these two algorithms are in good consistency. In our previous studies, bioinformatics could screen genes and construct features to predict the prognosis of prostate cancer, as well as explore molecular mechanisms of prostate cancer development [25–27]. The radiomics-based survival analysis performed well in predicting the prognosis for PCa patients, with the potential to optimize treatment protocols [28]. Radiomics combined with bioinformatics can help explore immunotherapy shortly.

The infiltration level of B cells in PCa is relatively higher compared with normal prostate tissue, suggesting that B cells can serve as a therapeutic target [29]. There are dispersed T-cell populations in both myeloid and blastic prostate cancers [30]. In metastatic castration-resistant PCa patients, Treg cell aggregation presents in the peripheral blood [31]. In the process of prostate carcinogenesis, M1 macrophages transform into the M2 phenotype, which promotes an immunosuppressive TME and thus tumor growth and metastasis [32]. It was proposed the higher the (M1 + M2)/M0 ratio, the worse the prognosis [33]. Consistently, in the low tumor purity group of our study, M0 cells were significantly decreased and Treg cells were increased considerably, who had a worse prognosis.

Two genes (FCER1G and OLR1) related to TPS were significantly associated with PCa progression and metastasis, as proposed in previous studies. For example, GLRX, SNAP23 and OLR1 are overexpressed, which is related to aggressive metastasis in breast cancer and prostate cancer tissues [34]. FCER1G is associated with TME in PCa, which may help to predict the prognosis of PCa [35]. It has been reported that metastasis-associated gene FCER1G was abundantly expressed in circulating tumor cells (CTCs) of a PCa patient who was sensitive to docetaxel, a chemotherapy agent [36]. There is a significant increase of SLAMF8 in PCa tissues, both at the RNA and protein levels. It is an important metastatic marker worthy of further study.

Significant variations in the immune microenvironment among various tumor purities were observed through the process of enrichment analysis. Low purity tumor exhibits increased infiltration of immune cells and a negative prognosis. Meanwhile, we discovered that hub genes were primarily concentrated in primary immunodeficiency disorder. Accordingly, metastasis of prostate cancer may be linked to immunosuppressive conditions caused by immune microenvironment disorders. Research findings in different types of malignancies strongly support this new perspective, such as non–small cell lung cancer (NSCLC) and melanoma patients with liver metastasis [37], renal cell carcinoma [38], lung cancer [39].

This study has several limitations. Firstly, tumor purity was calculated based on only one set of TCGA transcriptome data. Our finding needs to be validated using more data sets and multiple algorithms. Secondly, this is a retrospective study. A prospective evaluation would enhance the robustness of our findings.

## Conclusions

This study revealed that TPS can predict DMFS in PCa patients. Low TPS may result in better outcomes for patients with PCa due to a potential relationship between tumor immunity and tumor purity. Notably, TPS and nomogram models could have potential value in the prognostic stratification of PCa. Immune suppression may be an important mechanism for prostate cancer metastasis. Our study provides an essential clue for the clinical therapeutics of PCa.

### Supplementary Information

**Additional file 1: Table S1.** The results of tumor purity.

**Additional file 2: Table S2.** The results of WGCNA analysis.

## Data Availability

The data sets supporting the conclusions of this article are available in the TCGA repository, (https://portal.gdc.cancer.gov/), and GEO repository (https://www.ncbi.nlm.nih.gov/geo/).

## Abbreviations

PCa: Prostate cancer
TCGA: The Cancer Genome Atlas
PPI: Protein–protein interaction
GO: Gene Ontology
KEGG: Kyoto Encyclopedia of Genes and Genomes
DO: Disease Ontology
GSEA: Gene Set Enrichment Analysis
HPA: Human Protein Atlas
TPS: Tumor purity score
GSE: Gene Expression Omnibus Series
DMFS: Distant metastasis‐free survival
TH: T-helper lymphocytes
TNM: Tumor, node, metastasis
WGCNA: Weighted gene co-expression network analysis
LASSO: Least absolute shrinkage and selection operator
ROC: Receiver operating characteristics curve
GEO: Gene Expression Omnibus
NCBI: National Center for Biotechnology Information
NCI: National Cancer Institute
NHGRI: National Human Genome Research Institute
PSA: Prostate-specific antigen
MET: Metastasis
STRING: Search Tool for the Retrieval of Interacting Genes
PRAD: Prostate Adenocarcinoma
ssGSEA: Single-sample GSEA
GSVA: Gene Set Variation Analysis
DEGs: Differentially expressed genes
FDR: False discovery rate
AUC: Area under the curve
NNE: Nearest neighbor estimation
CI: Confidence interval
HR: Hazard ratio
BP: Biological process
CC: Cellular component
MF: Molecular functions
IHC: Immunohistochemistry
TME: Tumor microenvironment
CTCs: Circulating tumor cells
